# Compensator‐based intensity‐modulated radiation therapy for malignant pleural mesothelioma post extrapleural pneumonectomy

**DOI:** 10.1120/jacmp.v9i4.2799

**Published:** 2008-10-29

**Authors:** Khosrow Javedan, Craig W. Stevens, Kenneth M. Forster

**Affiliations:** ^1^ Radiation Oncology H. Lee Moffitt Cancer Center and Research Institute, and Radiation Oncology Tampa Florida U.S.A.; ^2^ H. Lee Moffitt Cancer Center and Research Institute at the University of South Florida Tampa Florida U.S.A.

**Keywords:** malignant pleural mesothelioma, compensator‐based IMRT, SMLC IMRT, plan conformality, quality assurance

## Abstract

The present work investigated the potential of compensator‐based intensity‐modulated radiation therapy (CB‐IMRT) as an alternative to multileaf collimator (MLC)–based intensity‐modulated radiation therapy (IMRT) to treat malignant pleural mesothelioma (MPM) post extrapleural pneumonectomy.

Treatment plans for 4 right‐sided and 1 left‐sided MPM post‐surgery cases were generated using a commercial treatment planning system, XIO/CMS (Computerized Medical Systems, St. Louis, MO). We used a 7‐gantry‐angle arrangement with 6 MV beams to generate these plans. The maximum required field size was 30×40 cm. We evaluated IMRT plans with brass compensators (•Decimal, Sanford, FL) by examining isodose distributions, dose–volume histograms, metrics to quantify conformal plan quality, and homogeneity. Quality assurance was performed for one of the compensator plans.

Conformal dose distributions were achieved with CB‐IMRT for all 5 cases, the average planning target volume (PTV) coverage being 95.1% of the PTV volume receiving the full prescription dose. The average lung $V_{20}$ (volume of lung receiving 20 Gy) was 1.8%, the mean lung dose was 6.7 Gy, and the average contralateral kidney $V_{15}$ was 0.6%. The average liver dose $V_{30}$ was 34.0% for the right‐sided cases and 10% for the left‐sided case. The average monitor units (MUs) per fraction were 980 MUs for the 45‐Gy prescriptions (mean: 50 Gy) and 1083 MUs for the 50‐Gy prescriptions (mean: 54 Gy).

Post surgery, CB‐IMRT for MPM is a feasible IMRT technique for treatment with a single isocenter. Compensator plans achieved dose objectives and were safely delivered on a Siemens Oncor machine (Siemens Medical Solutions, Malvern, PA). These plans showed acceptably conformal dose distributions as confirmed by multiple measurement techniques. Not all linear accelerators can deliver large‐field MLC‐based IMRT, but most can deliver a maximum conformal field of 40×40 cm. It is possible and reasonable to deliver IMRT with compensators for fields this size with most conventional linear accelerators.

PACS numbers: 87.56.ng, 87.56.N, 87.55.D, 87.55.dk

## I. INTRODUCTION

Treating malignant pleural mesothelioma (MPM) post surgery requires very large fields. The present paper addresses intensity‐modulated radiation therapy (IMRT) plans with solid modulators for the large fields required to treat MPM post surgery, given that these plans first closely achieved the prescription dose objectives, passed quality assurance (QA), and could be safely delivered.

Malignant pleural mesothelioma is a fatal aggressive cancer of the pleura and a large complex target volume. Reports show that the incidence of MPM is increasing globally, with 2000 new cases annually in the United States.^(1)^ Increased incidence of mesothelioma is strongly associated with exposure to asbestos, which is most commonly used in Western industrial societies; more men than women are affected.^(2)^ In 2003, the Surveillance, Epidemiology, and End Results Program of the U.S. National Cancer Institute projected the total number of MPM cases in American men to be approximately 71 000 by the year 2054.^(3)^ Because of the predicted numbers of new cases, the National Cancer Institute is sponsoring clinical trials designed to seek new treatment modalities.

Traditionally, radiation therapy treatment techniques for MPM used external‐beam radiation with a combination of photon and electron beams^(4)^ ^,^ ^(5)^ and intraoperative brachytherapy with postoperative mixed photon irradiation.^(6)^ Normal tissue was spared using photon and electron blocks for external‐beam treatments. Various dose regimens have been prescribed for palliation and local control of this disease, ranging from 30 Gy^(7)^ to a median dose of 36 Gy^(8)^ (palliation) and 54 Gy (45 – 54 Gy, local control)^(9)^ administered to the hemithorax. The latter treatment showed improved local control with acceptable toxicity. This finding seems to demonstrate that a sufficient dose was achieved for palliation and local control of the disease with the conventional techniques. But the published literature lacks metrics, including dose–volume histograms (DVHs), which have increasingly become a crucial part of plan review and comparisons complementing isodose distribution in transverse and orthogonal planes.

Radiation oncologists often use information from computed tomography (CT), magnetic resonance, and positron‐emission tomography imaging to accurately delineate the target and organs‐at‐risk (OARs) volumes so as to prescribe and quantify the dose to these sensitive overlapping structures. The use of IMRT allowed for further dose escalation to large target volumes while maintaining tolerance doses to abutting radiation‐sensitive structures.^(10)^

Postoperative IMRT for MPM has shown the most promising early local control of this disease.^(^ ^11^ ^–^ ^13^ ^)^ Current techniques often couple IMRT from a specific treatment planning system with specific beam delivery and verify systems. Stevens et al.^(14)^ found that Corvus, Pinnacle, and Eclipse treatment planning systems were all capable of generating acceptable IMRT plans for MPM after extrapleural pneumonectomy (EPP). Authors compared treatment planning systems and found that the early plans with Corvus had the largest number of monitor units (2786 MUs) and segments (1050 segments), and that a newer version of Eclipse had the least number of MUs (1813 MUs) and segments (173 segments).

Delivery of large IMRT fields with a multileaf collimator (MLC) is limited by MLC design.^(15)^ For example, the Siemens Oncor machine (Siemens Medical Solutions, Malvern, PA) with 82‐leaf Optifocus MLC system allows for a maximum IMRT field size of 22×40 cm. The MLC carriage‐over‐travel distance past the central axis is limited to 10 cm. Even though a field size of 24×40 cm can be accommodated, given that the smallest segment size can be set to 2×2 cm, larger IMRT field widths are required to treat MPM. To overcome the MLC field size limitations, treatments with multiple isocenters have been proposed by other investigators.^(16)^ We investigated a compensator‐based IMRT (CB‐IMRT) technique with a single isocenter to treat MPM post surgery.

It is essential that the modulator (MLC or solid brass compensator) reproduce the intended fluence map. For 3 of 4 right‐sided cases, a number of IMRT fields required a minimum field width of 26 cm. These cases were good candidates for CB‐IMRT delivery (which has no IMRT field size limit, and for which a maximum conformal field size of up to 40×40 cm is possible) on the Siemens Oncor machine. Intensity‐modulated radiation therapy with compensators has been successfully used for more than a decade.^(17)^ ^,^ ^(18)^ The CB‐IMRT technique offers continuous intensity modulation. Compensators deliver the intensity‐modulated dose in static form to all points within a field relatively instantaneously where the beam‐on time depends on the machine dose rate.

To investigate the potential of very large field CB‐IMRT for MPM, the goal was to create plans to be delivered on our Siemens Oncor treatment machine that closely achieved the prescription dose objectives for MPM post surgery and that produced manageable modulators.

## II. MATERIALS AND METHODS

All data sets acquired for this test study came from patients who underwent surgery before simulation.

### A. Surgery

The EPP procedure involves removal of the ipsilateral lung (remove motion) and hemidiaphragm resection, with subsequent reconstruction using polytetrafluoroethylene fabric. Mediastinal lymph nodes dissection is also performed, as is a chest wall resection and reconstruction. To assist the radiation oncologist with the contouring, the surgeons place clips to identify the entire outline of the resected hemidiaphragm and the resected margins. (These areas are otherwise difficult to identify postoperatively.) The simulation for treatment planning occurs 6 – 8 weeks post surgery.

### B. Simulation

Surgical scars were typically wired and then covered with bolus (7 cm wide, 0.50 cm thick) extended 4 cm proximally and distally over the scar. Patients were immobilized supine on the CT couch using a wing board (Med‐Tec, Orange City, IA) in combination with a vacuum bag and the T‐bar system indexed to the couch top. The T bar helps support the arms up and out of the radiation field. Radio‐opaque ball bearing markers were taped to the anterior and lateral sides of the patient for treatment planning and as a setup reference.

Simulation CT slice thicknesses were typically 5 mm (no larger). At least 100 transverse slices were acquired and transferred to the treatment planning system.

### C. Contours

The use of IMRT required contouring of the clinical target volumes (CTVs), which included the tumor bed (post EPP) and the regions at risk for seeding of disease. The CTV extended from T1 to L3 (from apex of thorax to inferior pole of kidney). The contours for the contralateral lung, kidneys, heart, liver, esophagus, small intestine, spinal cord, and skin were drawn on every slice (a time‐consuming process). The three dimensional auto margin functions were used to create a PTV0.5 (CTV + 0.5 cm) and cord avoidance (cord + 0.5 cm) structure. Avoidance and boost structures were drawn manually. To account for uncertainties in contouring the CTV, superior and inferior margins were set 1 cm above and 1 cm below the most superior and most inferior surgical clips. The anterior, posterior, and lateral margins were defined by adding 0.75 cm to violated spaces clipped at the skin.

### D. Treatment planning

Coplanar IMRT beams (6 MV) aimed at the center of the planning target volume (PTV) were designed. The first gantry angle was 180 degrees, and the remaining 6 angles were at about 30‐to 45‐degree intervals, excluding the anterior–posterior field. These were selected using a beam's eye view tool to minimize entrance and limit exit doses to the contralateral lung. Gantry angles were adjusted as much as possible while obtaining the desired dose distributions. We noticed that liver position varied in the superior–inferior direction with respect to CTV position for these cases. Planning right‐sided cases with sufficient CTV coverage, given the extent of liver in the radiation field, was more challenging because of the competing dose constraints of these structures.

### E. IMRT plans

Seven fields (gantry angles), A – G, were selected to conform to PTV using MLC. Fields A – D were split to keep the compensator weights manageable, resulting in additional fields A1 – D1. Fields A and A1 were split in the inferior–superior direction with at least a 2‐cm overlap margin. The PTV contour seen by each field was also edited and MLC‐conformed to the new split contour shapes. The same procedure was repeated for fields that needed to be split. The fields were split at varying distances from central axis to help reduce possible high dose at the junction area.

### F. IMRT prescription page

Target and OARs were set up in the IMRT prescription page according to the prescription guidelines shown in Table 1. Deviation from goal doses for the minimum and maximum PTV coverage was given the highest penalties. Liver, kidneys, contralateral lung, and heart were given higher overlap priorities than were the other OARs. Maximum dose to the liver was set to the maximum PTV dose with relaxed penalty, but certain percentages of liver and contralateral lung volumes were restricted to very low doses with very high penalties. The point was to achieve the suggested prescription dose objectives to these structures.

**Table 1:** Dose–volume guidelines for the target and organs at risk (OARs)^a^

*Target or OAR*	*Dose–volume guideline*
Clinical target volume	$V_{100}>98\%$
Planning target volume	$V_{100}>95\%$
Contralateral lung	$V_{20}<4\%$
Mean lung dose	6−8 Gy or ALARA
Spinal cord	Less than 10%>45Gy
	0.0%>50Gy
Heart	$V_{45}<50\%$
Liver	$V_{30}<30\%$ or ALARA
Right kidney	$V_{15}<20\%$
Left kidney	$V_{15}<20\%$
Esophagus	$V_{55}<30\%$

a The planning target volume is the clinical target volume plus 0.5 cm. The $V_{100}$ is the volume receiving 100% of the prescribed dose. Contralateral lung $V_{20}$ is the volume of lung receiving 20 Gy. Lung mean lung dose and liver $V_{30}$ are kept as low as reasonably achievable (ALARA).

### G. Compensator plans

#### G.1 Treatment planning strategy

**Figure 1:**
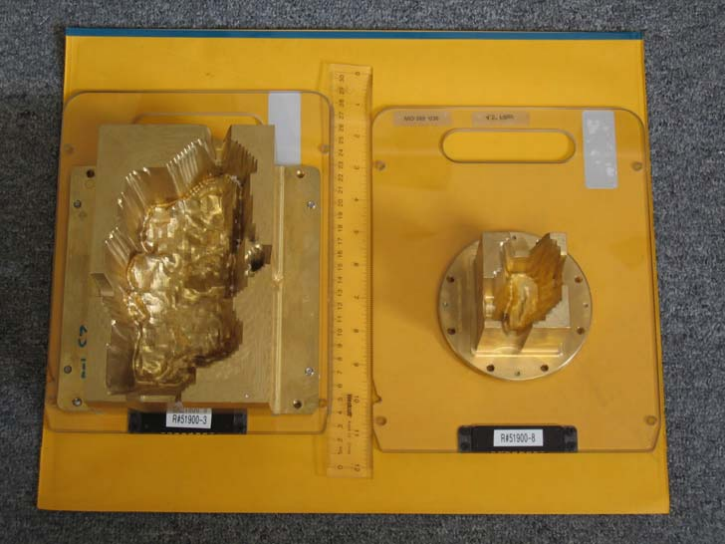
Modulators from •Decimal mounted on the Siemens coded trays. One of the large compensators (11.3 kg) from the initial 7‐field plan that did not require extensive blocking within the field is shown next to one of the 7‐field IMRT modulators for prostate (1.8 kg).

Our CB‐IMRT plan strategy process started with an arrangement of 5 non‐coplanar fields for the left‐sided case and arrangements of 7 coplanar fields for the right‐sided cases, which resulted in acceptable dose distributions, but very heavy modulators (weight up to 15.9 kg). An 11.3‐kg compensator from the 7‐field arrangement is shown in Fig. 1. These large fields were then split, which resulted in 7‐gantry 14‐coplanar fields with field junction overlap matched at the central axis, using modulators 7.62 cm thick that yielded acceptable dose distributions with 125% hot spots in the junction area. The average compensator weight was 8.2 kg (range: 6.3 – 9.1 kg). We did not achieve the plan objectives if all beams were modulated with the brass modulators (5.08 cm maximum thickness), even with MLC blocking. The outcome was a 7‐gantry 11‐coplanar field arrangement with modulators (5.08 cm and 7.62 cm maximum thickness) and field junction overlap at varying distance from the central axis. The median compensator weight was 7.5 kg (5.2 – 10.0 kg).

All CB‐IMRT plans were generated using MLC blocking that conformed the beam to the PTV plus a 0.5‐cm block margin. We found it desirable to plan the compensator fields with MLC blocking advantages. In addition, individual MLC leaves can be edited to further enhance the distribution even after the compensators are generated. The effective attenuation coefficient (EAC) values were assigned for all fields. We used compensators (7.62 cm maximum thickness) with an intensity modulation range of 8% – 100% for fields that contained significant parts of the contralateral lung or liver. These large fields would produce heavy modulators, and thus were divided. We used compensators (5.08 cm maximum thickness) with an intensity modulation range of 20% – 100% for fields that contained no significant parts of lung or liver. The only remaining lung had to be spared. We tried to keep the 10‐Gy isodose line outside the contralateral lung and kidney volumes to maintain prescription guidelines to these structures. All IMRT plans were calculated using superposition with heterogeneity corrections applied.

Dose distributions and DVHs were reviewed by the radiation oncologist. Each field was set up in the IMPAC Record and Verify system (IMPAC Medical Systems, Sunnyvale, CA) with its unique accessory tray code (S2N01–S2N18). Two coded trays are shown in Fig. 1. Compensator thickness files were electronically sent to •Decimal for fabrication; the modulators were returned within 24 hours.

#### G.2 Compensator thickness file

The brass thickness *t* (*i*,*j*) can be calculated as a simple exponential attenuation equation for an array of values each representing the filter thickness for each ray line at (*i*, *j*) as in equation 1:

(1) $$t(i,j)=-\ln\;[\text{Trans}(i,j)]/\mu^{\text{eff}},$$

where Trans(*i*, *j*) is the transmission for each ray line throughout the compensator, μ^eff^ is the EAC for solid brass compensator under broad‐beam geometry. du Plessis et al.^(19)^ showed that the absorbed dose varies exponentially as a function of absorber thickness on the beam axis at any depth in water for any material. These authors showed that EAC for brass can vary by as much as 13% over a depth range of 4 – 39 cm. The measurement showed that EAC values decreased with increasing field size, depth, and thickness as beam hardening and more scatter for larger field sizes contributed to dose at the given depth.

Beam divergence and beam hardening is taken into account by the treatment planning system's dose computation. Mean energy of the beam increases after the beam passes through the modulator. Jiang and Ayyangar^(20)^ showed that beam hardening resulted in greater change (sparing) in surface dose than in dose at depth with respect to maximum dose. Authors showed that for a 6‐MV 10×10 beam, a 10% maximum dose reduction occurred in surface dose and a 3% dose increase occurred in percent depth dose at 10 cm depth for Cerrobend slab 5 cm thick ($p=9.76g/{\text{cm}}^{3}$).

#### G.3 Safety considerations

We considered limiting the maximum compensator weight to a level that therapists felt comfortable safely handling. We had the therapists try the 10‐kg and 15.9‐kg compensators. The therapists who could easily handle the 45‐degree solid wedge (which is 6.1 kg) were able to insert the 10‐kg modulators into the wedge slot with ease; the 15.9‐kg modulators posed much more of challenge for most of the therapists. The manufacturer's recommended weight limit for the Siemens Oncor block tray accessory is 15.9 kg; however, we do not have the weight‐limit information for the wedge tray slot accessory. We tried to exercise safety in handling the relatively heavy modulators.

Modulators were loaded with the gantry set at 90 degrees or 270 degrees. The delivery was such that no loaded compensator field crossed over the patient at any time, and for patient safety, plans were designed without the anterior–posterior field.

### H. Plan evaluation

We used two‐dimensional isodose distributions (axial, sagittal, and coronal planes) to visually inspect the target coverage. Quantitative techniques such as DVH analysis and other indices were used to evaluate the plans. Table 2 shows the fraction of PTV volume receiving 100% of the prescribed dose in grays (PTV $V_{100}$); high dose in grays to 5% of PTV volume (PTV $D_{05}$); conformation number (CN), an index proposed by van't Riet et al.,^(21)^ which takes into account the quality of coverage of the target and the volume of healthy tissue receiving at least the prescribed dose; and the homogeneity index (HI) as the ratio between maximum dose and prescription dose within the target. In addition, contralateral lung $V_{20}$, $V_{5}$, and mean lung dose (MLD); $V_{30}$ for liver; $V_{45}$ and $V_{50}$ for heart; $V_{15}$ for contralateral kidney; and maximum cord dose and dose to 10% of cord $\left(D_{10}\right)$ were used to score OAR protection. Treatment efficacy with compensators in terms of MUs per field, total MUs, and beam‐on time per daily fraction and per total treatment time are reported.

**Table 2:** Plan values^a^

*Plan ID*	$\text{PTV}\;V_{100}\;(\%)$	$\text{PTV}\;D_{05}\;(\text{Gy})$	(${\text{TV}}_{\text{RI}}/\text{TV})$	(${\text{TV}}_{\text{RI}}$/$V_{\text{RI}}$)	*CN*	*HI*
1RC	95	52	0.95	0.96	0.92	1.3
1RM	95	55	0.95	0.96	0.91	1.4
2RC	95	54	0.95	0.82	0.78	1.5
3RC	95	54	0.95	0.90	0.86	1.2
4RC	95	56	0.95	0.93	0.88	1.2
5LC	97	52	0.97	0.85	0.83	1.2
5LM	98	55	0.98	0.81	0.80	1.5

a Plan 1RC (plan 1, right‐sided case, with compensators) shows that 95% of the planning target volume (PTV) received 100% $\left(V_{100}\right)$ of the prescribed dose of 45 Gy. The high dose to 5% of the PTV volume is 52 Gy for the compensator plan and 55 Gy for the multileaf collimator (MLC) plan 1RM (plan1, right‐sided case, with MLC).

PTV=planning target volume; TV=target volume; RI=reference isodose line; CN=conformation number; HI=homogeneity index; $V_{100}=\text{volume}$ receiving 100% of the prescribed dose; $D_{05}=\text{high}$ dose to 5% of the volume; plan ID key: plan number (1 – 5), right‐ or left‐sided (R, L), compensator or multileaf collimator (C, M).

### I. Quality assurance

We used multiple measurement techniques to perform IMRT QA. Dose distributions for one of the compensator plans were recalculated for three QA phantoms. This repetition provided the reference for comparison with absolute point dosimetry measurement using a calibrated ion chamber, single coronal field using absolute dose distributions measurement with the Map CHECK diode array device (Sun Nuclear Corporation, Melbourne, FL), and composite dose distributions at 4 transverse film planes irradiated simultaneously with true gantry angles incident on the phantom. We registered extended dose range (EDR2) films to the plan and analyzed them using the RIT 113 film dosimetry system (RIT, Denver, CO).

## III. RESULTS

**Figure 2:**
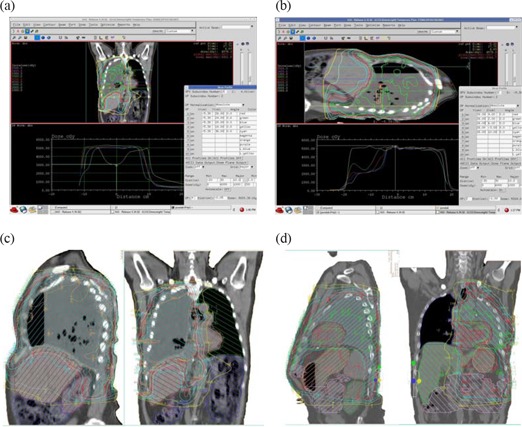
Dose distributions in the coronal and sagittal planes. (a) Sagittal profile of a right‐sided case shows the profiles across the coronal plane at isocenter, ±2cm from isocenter, and 6 cm inferior and 12 cm superior to isocenter. The profile 6 cm inferior to isocenter shows the degree of liver‐sparing in this plane. (b) Coronal profile of a right‐sided case shows the profiles at isocenter, ±2cm from isocenter, and 5 cm posterior and 8 cm anterior to isocenter plane. (d) Dose distribution for the left‐sided case is shown.

**Figure 3:**
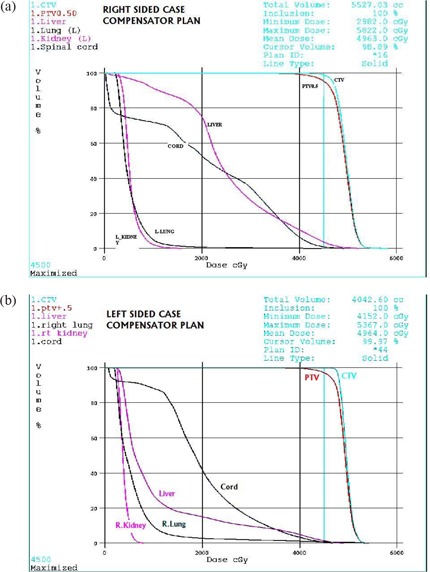
Dose–volume histograms for the planning target volume (PTV), clinical target volume (CTV), and the liver, lung, kidneys, and spinal cord for (a) a right‐sided case, and (b) the left‐sided case.

Fig. 2(a)–(d) shows isodose distributions for right‐sided and left‐sided cases. Notice that the 10‐Gy isodose line is kept outside the contralateral lung. Fig. 2(a),(b) shows dose distributions in the coronal and sagittal planes, and dose profile at various distances from the central axis in those planes. Fig. 2(a) shows the coronal dose distributions for a right‐sided case. The flat dose profiles show that dose varies 2% across the coronal plane and 6% along the plane. Fig. 2(b) shows the dose distributions for the right‐sided case in the sagittal plane. The profile throughout the field junction shows 118% hot spots. Fig. 2(c) shows the 50‐Gy prescription dose distributions for a right‐sided case. Fig. 2(d) shows the dose distributions for the left‐sided case. Fig. 3(a),(b) shows DVHs for one of the right‐sided and the left‐sided MPM case.

All plans conformed to 99.2% of CTV volume and 95.5% of PTV volume achieving the prescription dose. Lung $V_{20}$ was less than 2%, and MLD ranged between 5 Gy and 8 Gy for all plans. All lung MLDs and $V_{5}$s were below the range at which pneumonitis was no longer reported to have been observed by other investigators.^(22)^ The average value for liver $V_{30}$ was 34%.

To compare IMRT treatment parameters with segmented MLC (SMLC) and compensator delivery on the Siemens machine, we used the same field configuration in planning 2 cases with SMLC IMRT. Large optimized fluence had to be segmented with a minimum MLC segment size greater than 2 cm^2^ (because of IMRT field size limitations) to produce deliverable plans. More desirable and better dose delivery resolution was not possible in this case. In Table 2, note that $\left({\text{TV}}_{\text{RI}}/V_{\text{RI}}\right)$ is worst for plan 5 with MLC (5LM). As compared with compensator plan 5LC, this plan shows more non‐target tissue receiving the prescription dose. One possible explanation may be that plan 5LM needed to have a smaller segment size, and thus higher dose sculpting power, to block portions that needed to receive less dose. This plan also had higher HI than did the compensator plan. Table 2 also shows MLC parameters such as total number of segments and MUs, and total treatment time. Total MUs were 1993 MUs with 244 segments for plan 1RM (Table 2). Treatment time per beam can be seen to be significantly shorter for compensators. The total MUs were doubled for SMLC. Total treatment time was slightly shorter for compensator delivery, and yet comparable with automated SMLC delivery, as shown in Table 3.

**Table 3:** Plan delivery values^a^

*Plan ID*	*Average MUs per field*	*Total MUs*	*Segments*	*Average beam‐on time per field (s)*	*Total treatment time (min)*
1RC	89	976	—	18	33
1RC^b^	98	1083	—	23	34
1RM	181	1993	244	198	36
2RC	87	962	—	18	33
3RC	94	1040	—	19	35
4RC	97	1039	—	19	35
Avg. RC	92	1004	—	18.5	34
5LC	80	882	—	16	30
5LM	164	1801	193	126	35

a The average beam‐on time per field for compensator plans 1RC – 4RC and 5LC was calculated based on 300 cGy/MU at the central axis. The number of segments are shown for the multileaf collimator plans 1RM and 5LM. Total treatment time for compensator delivery includes entering the treatment room to replace the compensator for each field. Plan ID key: plan number (1 – 5), right‐ or left‐sided (R, L), compensator or multileaf collimator (C, M).

b For 50 Gy prescription dose.

Table 3 shows the number of MUs for the 5 compensator and 2 MLC plans. Our plans for 4 right‐sided and 1 left‐sided case resulted in an average of 980 MUs (range: 882 – 1040 MUs) per daily fraction. The average daily delivery time was 33 minutes, which included entering the room to check the isocenter and to replace the compensators for all fields.

### A. QA results

**Figure 4:**
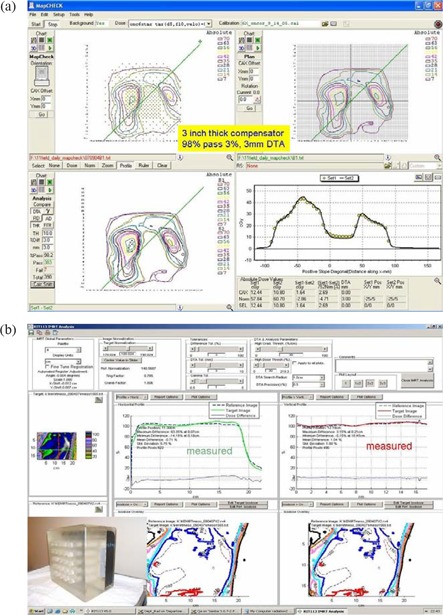
(a) The calculated and measured isodose distributions in the coronal plane for one of the compensator fields is shown in the top right and top left quadrants. At the bottom left, the overlaid absolute dose distributions are seen. Overlaid oblique profiles is shown in the bottom right quadrant. DTA=distance to agreement. (b) The cube phantom is shown at the lower left. The RIT film dosimetry system (RIT, Denver, CO) analysis window shows good agreement between the measured and calculated dose distributions.

The measured absolute dose in water and in a cube phantom for the composite plan agreed within 3% of the calculated dose. Fig. 4(a) shows one of the individual fluence maps from Map CHECK. At least 95% of the measured and calculated isodose distributions were found to be in agreement within 3% and 3 mm distance to agreement for all individual compensator fields. The large EDR2 film dosimetry showed 117% hot spots in the junction area, consistent with the predicted value. Fig 4(b) shows the QA results using the cube phantom. The measured orthogonal profiles were extracted, which showed good agreement with the calculated profiles.

## IV. DISCUSSION

Although the words “compensator” and “solid modulator” were used interchangeably throughout this paper, the intention was to refer to the same device that modifies the intensity of the beam. The goal of the present study was to devise a CB‐IMRT technique for MPM post surgery that met the prescription objectives and could be safely delivered. The results reported here are based on the reference dose of 45 Gy. We also tested the technique for a higher prescription dose and found that the results also apply to the prescription dose of 50 Gy. We re‐optimized plan IRC (planl, right‐sided case, with compensators) for a higher prescription dose of 50 Gy. Fig. 2(c) shows the dose distribution. We achieved the prescription objectives with at least 95% of the PTV volume receiving 50 Gy, with a mean dose of 54 Gy. Liver $V_{30}$ was less than 36%, and contralateral lung MLD and $V_{20}$ were 5.7 Gy and 1% respectively. No portion of cord received 50 Gy. The total MUs for the 50‐Gy plan for the right‐sided case were 1083 MUs.

We used CN as one of the metrics to quantify conformal plan quality. The CN is defined in equation 2:

(2) $$\text{CN}=({\text{TV}}_{\text{RI}}/\text{TV})*({\text{TV}}_{\text{RI}}/V_{\text{RI}}).$$

We calculated the DVHs for all tissue and non‐tissue (unspecified tissue not contoured). The target volume covered by the reference isodose line, ${\text{TV}}_{\text{RI}}$, was calculated. The first term in equation 1 takes account only of quality of target coverage. This value for right‐sided plans was 0.95 for compensator plans (as in Table 2). The second term describes the ratio of the PTV volume that received the prescription dose to the volume of the reference isodose line. All non‐target tissue and non‐tissue volumes covered by the reference isodose line were summed. The average values for the 4 right‐sided cases were 0.90 for compensator plans and 7.2% of non‐target tissue. For the left‐sided case with compensators, 12.3% received the prescription dose. If the CN value is 1, the target conformity is 100% and the dose must fall rapidly outside the large PTV. As indicated earlier, part of the liver and kidney volumes fell inside the PTV because of overlap. In fact, about 10% of liver volume received the reference dose.

## V. CONCLUSIONS

We found that CB‐IMRT with 7 gantry angles produced dosimetrically acceptable plans for a single isocenter, without the need to match electron fields; however, it produced heavy modulators. The same gantry angles with 11 coplanar 6‐MV IMRT fields produced acceptable conformal plans and closely achieved the prescription dose objectives. The resulting modulators with an equivalent field size of 26 cm^2^ were easier to manage. Total treatment time for manual CB‐IMRT delivery was comparable to that with automated SMLC delivery. For MPM, CB‐IMRT showed acceptably conformal dose distributions confirmed by multiple measurement techniques. Not all linear accelerators can deliver large‐field SMLC‐based IMRT with a single isocenter, but most can deliver a maximum conformal field size up to 40×40 cm. It is possible and reasonable to deliver IMRT with compensators for fields this size with most conventional linear accelerators. The ability to deliver IMRT with solid modulators adds an option to existing linear accelerators to treat large target volumes, as is the case for MPM post EPP.

## ACKNOWLEDGMENTS

Our research was supported by •Decimal Inc. The authors thank Richard Sweat, Chris Warner, Ken Cashon, Lisa Cashon, and the •Decimal team for their courteous support. We have no commercial interest in •Decimal, and we declare no conflict of interest with •Decimal Inc.

The authors also thank Amarjit Saini for a review of the manuscript and for constructive remarks. The authors also thank Spencer Marshall at CMS, Inc., for technical support.
